# Iatrogenic fracture during shoulder dislocation reduction: characteristics, management and outcomes

**DOI:** 10.1186/s40001-021-00545-3

**Published:** 2021-07-12

**Authors:** Xiaohui Pan, Yong Yao, Hongyong Yan, Jun Wang, Lei Dai, Xincong Qu, Zuyi Fang, Feng Feng, Yan Zhou

**Affiliations:** 1 Department of Orthopedics, Luotian County People’s Hospital, Luotian, 438600 Hubei People’s Republic of China; 2 Department of Orthopedics, The Central Hospital of Enshi Autonomous Prefecture, Enshi Clinical College of Wuhan University, Enshi, 445000 Hubei People’s Republic of China; 3 Department of Orthopedics, Jiangxia District Hospital of Traditional Chinese Medicine, Jiangxia, 430200 Hubei People’s Republic of China; 4 Department of Orthopedics, Huangshi Second People’s Hospital, Huangshi, 435000 Hubei People’s Republic of China; 5 Department of Orthopedics, Huangmei County Hospital of Traditional Chinese Medicine, Huangmei, 438500 Hubei People’s Republic of China; 6 Department of Orthopedics, Renmin Hospital of Wuhan University, #238 Jiefang Road, Wuhan, 430060 Hubei People’s Republic of China

**Keywords:** Proximal humerus, Iatrogenic injury, Open reduction, Internal fixation, Outcome

## Abstract

**Background:**

Shoulder dislocation and the cases of iatrogenic fractures during manual reduction are becoming increasingly common. The aim of this study was to investigate the characteristics, management, and patient outcomes of iatrogenic proximal humeral fracture during the manual reduction of shoulder dislocation.

**Methods:**

A retrospective and multi-center study was performed to identify all patients presenting with shoulder dislocation from January 2010 to January 2020. The sex and age of patients, associated injuries, first-time or habitual shoulder dislocation, type of anesthesia, time from injury to revision surgery, and functional outcomes were analyzed.

**Results:**

A total of 359 patients with a mean age of 62.1 ± 7.3 years (range 29–86 years) were included. Twenty-one patients (female/male ratio 17:4) with an average age of 66.3 ± 9.7 years (range 48–86 years) were identified with a post-reduction iatrogenic fracture. Female cases with greater tuberosity fractures (GTF) were more likely than male cases to have iatrogenic fractures during reduction (*P* = 0.035). Women aged 60 years or older experienced more iatrogenic fractures during manual reduction (*P* = 0.026). Closed reduction under conscious sedation was more likely than that under general anesthesia to have iatrogenic fractures (*P* = 0.000). A total of 21 patients underwent open reduction and internal fixation (ORIF) when iatrogenic fractures occurred. The mean follow-up period was 19.7 ± 6.7 months (range 12–36 months). The mean Neer scores were 80.5 ± 7.6 (range 62–93), and the mean visual analog score (VAS) was 3.3 ± 1.5 (range 1–6). Significant differences were observed in the Neer score and VAS with the time (more or less 8 h) from injury to revision surgery (*P* < 0.05).

**Conclusion:**

A high risk of iatrogenic proximal humeral fracture is present in shoulder dislocation with GTF in senile females without general anesthesia. ORIF performed in a timely manner may help improve functional outcomes in the case of iatrogenic injury.

## Background

Shoulder dislocation, which accounts for more than 50% of joint dislocations in the body, is the most common and orthopedic emergency work that requires immediate treatment [1]. Manual reduction of the dislocated shoulder is a common procedure in the emergency department. However, this procedure is not without risks and can lead to serious complications. An unfortunate and difficult subset of these injuries includes iatrogenic or exacerbated fractures during reduction procedures of anterior shoulder dislocations. Elderly patients with first-time dislocation are found to be an important risk factor for this serious injury. Additionally, when effective anesthesia is not properly given during reduction, especially in elderly patients with osteoporosis, forced reduction is likely to cause combined injury, and eventually iatrogenic proximal humeral fractures occur [2, 3]. At present, most scholars believe that the causes of such complications are closely related to repeated rough manual reduction without pain relief and muscle relaxation. During manual reduction under general anesthesia (GA) in an ideal state of muscle relaxation, iatrogenic injuries are likely to be avoided, even by a junior emergency medicine physician [4, 5].

Proximal humerus fracture–dislocations are serious injuries of shoulder joints, and related iatrogenic injuries are not uncommon in the clinic. Management of these injuries may cause obvious damage to the blood supply of the head of the humerus and avascular necrosis of the humeral head easily occurs. The consequence can seriously hinder the function of the shoulder joint and easily incur medical disputes [6]. The doctor performing the emergency management of patients with shoulder dislocation must be meticulous during inspection before the reduction to further ensure safe and effective intervention [7, 8].

The purpose of this retrospective and multi-center study was to investigate the characteristics, management, and patient outcomes of iatrogenic proximal humeral fracture during the manual reduction of shoulder dislocation. This study further supplemented the factors related to iatrogenic injury, including the type of anesthesia, as well as the significance and clinical outcomes of revision surgery, some of which have not been seen or rarely reported in previous studies.

## Material and methods

### Research design and patient selection

This study is a retrospective and multi-center case series with prospectively gathered data from January 2010 to January 2020 in six hospitals in mainland China, including Renmin Hospital of Wuhan University, Luotian County People’s Hospital, The Central Hospital of Enshi Autonomous Prefecture, Jiangxia District Hospital of Traditional Chinese Medicine, Huangshi Second People’s Hospital, and Huangmei County Hospital of Traditional Chinese Medicine. The inclusion criteria were as follows: (1) 18 years or older, (2) anterior shoulder dislocation, and (3) diagnosis of simple shoulder dislocation or shoulder dislocation with greater tuberosity fracture (GTF). Radiographs of GTF were classified as Type 11-A1 according to the Orthopaedic Trauma Association classification, and as one-part or two-part GTF with shoulder dislocation according to the Neer classification [9]. The study excluded patients with associated brain injury, open abdominal injury, multiple fractures, vascular injuries, and inadequate preoperative or postoperative films. Data collected included the sex and age of patients, associated injuries, first-time or habitual shoulder dislocation, type of anesthesia, time from injury to revision surgery, and functional outcomes.

All manual reductions were performed under conscious sedation or GA by orthopedic surgeons in an operating or fracture reduction room. The radiographic evaluation included an anteroposterior view of the glenohumeral joint (anteroposterior oblique in neutral rotation) and a scapular-Y view or trauma variant (Velpeau axillary) with proper radiographic technique. Fracture classification was based on plain radiographs and determined by two experienced orthopedic surgeons. The reductions were confirmed immediately under the C-arm X-ray machine perspective.

### Revision surgery and follow-up

The patients underwent revision surgery with open reduction with internal fixation (ORIF) after iatrogenic proximal humerus fracture occurred. These patients were evaluated monthly after the operation, every 2 to 3 months after fracture union, and every 6 months 1 year later. At the final follow-up, the functional recovery was assessed using Neer’s criteria and the visual analog score (VAS) (0 = very satisfied, 10 = no satisfaction) [10, 11].

### Statistical analysis

Data were statistically analyzed using the SPSS 16.0 statistical software. Continuous variables, expressed as the mean ± standard deviation (SD) and categorical variables as number (*n*) and percentage (%) were compared by the Student’s *t* test to detect group differences. The qualitative data of groups were compared by the *χ*^2^ test. For all tests, *P* values < 0.05 were considered to be significant.

## Results

### Epidemiology

A total of 359 patients were enrolled in this study, including 183 women (51.0%) and 176 men (49.0%). The average age was 62.1 ± 7.3 years (range 29–86 years). One-hundred sixty (44.6%) patients had GTF on their initial radiographs, 87 (42.6%) were in women and 73 (46.6%) were in men. There was no correlation between sex and the occurrence of GTF (*P* = 0.985). Of the 87 female cases involving GTF, iatrogenic fractures occurred in 14 (19.2%) cases, while four (7.3%) out of 73 male cases had an iatrogenic fracture. Female cases with GTF were more likely than men to have iatrogenic fractures during closed reduction (*P* = 0.035). Meanwhile, no statistically significant difference was observed in iatrogenic fractures between male and female without GTF (*P* = 0.107, Table 1).

**Table 1:** Demographics and variables

Variables	Iatrogenic fractures	Non-iatrogenic fractures	*P* value
Gender, *n* (%)			0.005
Male	4 (19.0%)	172 (50.9%)	
Female	17 (81.0%)	166 (49.1%)	
Female age, *n* (%)			0.026
< 60 years	2 (11.8%)	65 (39.2%)	
≥ 60 years	15 (88.2%)	101 (60.8%)	
GTF, *n* (%)			0.035
Male	4 (22.2%)	69 (48.6%)	
Female	14 (77.8%)	73 (51.4%)	
Non-GTF, *n* (%)			0.107
Male	0	92 (46.9%)	
Female	3 (100%)	104 (53.1%)	
Number of dislocation, *n* (%)			0.425
First-time	21 (100%)	328 (97.0%)	
Habitual	0	10 (3.0%)	
Anesthesia, *n* (%)			0.000
GA	4 (19.0%)	217 (64.2%)	
Conscious sedation	17 (81.0%)	121 (35.8%)	
Neer score (mean ± SD)			0.000
< 8 h	85.0 ± 4.5		
≥ 8 h	74.4 ± 6.6		
VAS (mean ± SD)			0.002
< 8 h	2.5 ± 1.2		
≥ 8 h	4.4 ± 1.1		

In 183 female cases, iatrogenic fractures occurred in 15 (12.9%) of 116 women ≥ 60 years old while only two (3.0%) of 67 women < 60 years old presented with iatrogenic fractures. Women aged 60 years or older experienced more iatrogenic fractures during manual reduction (*P* = 0.026, Table 1).

### Types of anesthesia

In 359 total cases, 221 (61.6%) had manual reduction attempt under GA and 138 (38.4%) had such attempt under conscious sedation. Of 21 cases with iatrogenic fractures in this study, four underwent GA and 17 underwent conscious sedation during closed reduction. Closed reduction under conscious sedation was more likely than GA to have iatrogenic fractures (*P* = 0.000, Table 1).

### Injury types and damage factors

**Fig. 1:**
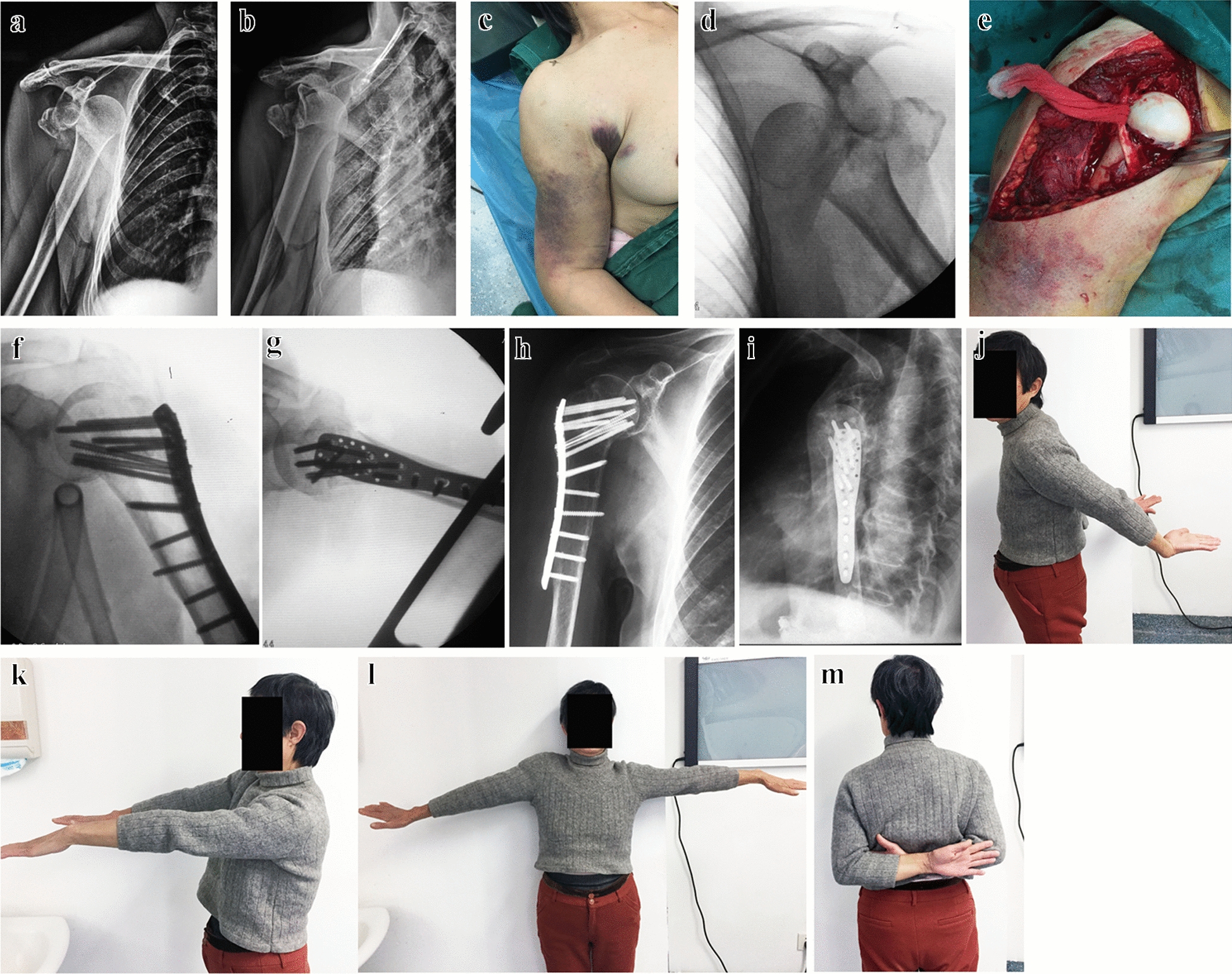
Iatrogenic fracture occurred and revision surgery. **a**, **b** Preoperative radiographs. **c** General appearance. **d** Iatrogenic fracture occurred. **e** Intra-operative situation. **f**, **g** C-arm fluoroscopy. **h**, **i** Postoperative radiograph at 12 months. **j**–**m** Range of motion at 12-month follow-up

**Fig. 2:**
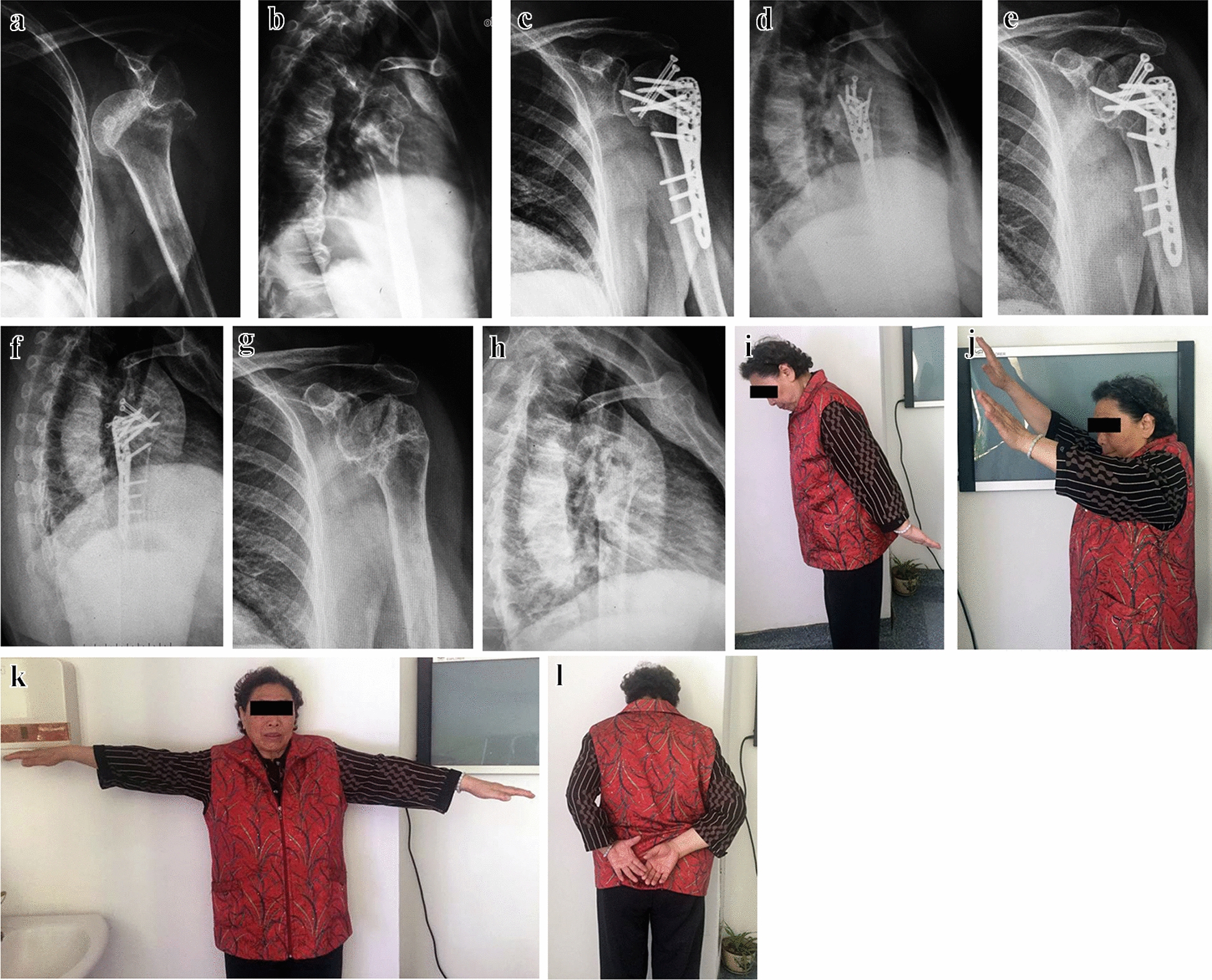
Iatrogenic fracture occurred and revision surgery. **a**, **b** Preoperative radiographs. **c**, **d** Postoperative radiograph after revision surgery. **e**, **f** Postoperative radiograph at 36 months. **g**, **h** Postoperative radiograph at 36 months after removing plate. **i**–**l** Range of motion at 36-month follow-up

Twenty-one patients (female/male ratio 17:4) with an average age of 66.3 ± 9.7 years (range 48–86 years) were identified with a post-reduction iatrogenic fracture. All these patients presented with first-time anterior shoulder dislocation, 18 patients (85.7%) were associated with GTF (Fig. 1) and two patients (9.5%) with Hill–Sachs lesion (Fig. 2). Post-reduction iatrogenic fracture was caused by low-energy falls (from a standing position) in 10 (47.6%) patients, high falling injury (from a considerable height or down-stairs) in eight (38.1%) patients, and motor vehicle accidents in three (14.3%) patients. Average time from injury to revision surgery (ORIF) was 10.5 ± 9.9 h (range 3–48 h).

### Clinical efficacy and complications

The mean follow-up period was 19.7 ± 6.7 months (range 12–36 months). Fracture union was achieved in these 21 cases. The mean time to union was 8.6 weeks (range 7–13 weeks). The mean neck–shaft angle at the final follow-up was 127.9° ± 9.2° (range 110°–142°). The mean Neer score was 80.5 ± 7.6 (range 62–93), and the mean VAS was 3.3 ± 1.5 (range 1–6). There were 9 patients with a treatment waiting time of more than 8 h (range 8–48 h), and the Neer score and VAS were 74.4 ± 6.6 (range 62–83) and 4.4 ± 1.1 (range 3–6), respectively. There were 12 patients with a treatment waiting time less than 8 h (range 3–7 h), and the Neer score and VAS were 85.0 ± 4.5 (range 79–93) and 2.5 ± 1.2 (range 1–5), respectively. Significant differences were observed in the Neer score (*P* = 0.000) and VAS (*P* = 0.002) with treatment waiting time (Table 1).

Two patients with brachial plexus nerve injury recovered 3 months after surgery. One patient presented superficial infection of the wound, which healed after debridement and perfusion drainage. Avascular necrosis of the humeral head occurred in three cases.

## Discussion

The purpose of this study was to assess the characteristics, management, and patient outcomes of iatrogenic proximal humeral fracture during the manual reduction of shoulder dislocation. We understood the characteristics of high-risk groups and high-risk factors for iatrogenic proximal humeral fracture during reduction and were guided in preliminary screening and targeted treatment during emergency work.

Besides young individuals, elderly women over the age of 50 years are prone to primary shoulder dislocation. This observation is highly consistent with the elderly population of osteoporotic proximal humerus fractures [12]. The risk of first-time shoulder dislocation combined with proximal humerus fracture in elderly patients is much higher than that in young and habitually dislocated patients. Research shows that iatrogenic fractures occur in about 80% of elderly women owing to age-related elements, such as postmenopausal osteoporosis [2, 13]. In the course of diagnosis and treatment, the neglect of these characteristics of senile shoulder dislocation will lead to serious complications [14]. In the present study, we found that the vast majority of these patients were elderly women with first-time anterior shoulder dislocation, and the highest priority for prevention should be given to elderly women because of their bone fragility.

The high-risk population of iatrogenic injury includes patients with anterior shoulder dislocation who are prone to proximal humeral fracture or with GTF during manual reduction. Considering our experience and literature review, we believe that the characteristics of high-risk groups are as follows: (1) elderly women over 60 years of age; (2) first-time anterior shoulder dislocation; (3) concomitant GTF or Hill–Sachs lesion [15, 16], and (4) displaced humerus head located below or medial to the coracoid process. Based on our observations, about 85.7% of the original dislocation types belong to the Neer one-part or two-part GTF with shoulder dislocation type. The fragment size, shape, and location of GTF may reveal diverse mechanisms and rapidity of injury, and the size of GTF is proportional to the incidence of iatrogenic humeral neck fractures [17, 18]. As to the type of analgesia/anesthesia adopted for shoulder reduction, several options are available, such as intra-muscular analgesics, intra-articular anesthesia, conscious or unconscious sedation, and GA. In the present study, we compared conscious sedation and GA during closed reduction and found that closed reduction under conscious sedation was more likely than that under GA to have iatrogenic fractures.

Currently, most scholars believe that the causes of such complications are related to repeated rough manipulations in patients without pain relief and muscle relaxation [19]. However, many scholars have reported that during manual reduction under GA in an ideal state of muscle relaxation, iatrogenic injuries are difficult to avoid, even by a senior orthopedic surgeon [6, 16]. These scholars proposed that the cause of iatrogenic injuries is related to the omission of unrecognized neck fracture before reduction [20]. Given the lack of additional pre-reduction imaging data in most cases, we were unable to understand whether occult anatomical neck fracture existed concomitantly. Whether the surgical or anatomical neck fracture was caused by improper manual reduction or inadvertent accompanying reduction, inappropriate treatment aggravated the displacement between the humeral head and shaft. The common consequence is the worsening of the fracture and dislocation type of the proximal humerus. In our enrolled cases, there was no description of the existence of occult fractures, or at least it was difficult to judge accurately on X-rays. A reasonable suggestion at this point is to run CT and MRI examinations of shoulder joints before the reduction to further ensure the absence of an occult fracture. The causes of iatrogenic proximal humerus fracture are associated with errors in diagnosis and/or treatment and should be classified as iatrogenic complications. Identifying the characteristics of iatrogenic injuries will help prevent similar mistakes in future work.

The overall incidence of iatrogenic proximal humeral fracture was 5.4%, but the incidence of GTF can increase to 26% [15]. We found that the affected patients were basically elderly women with first-time anterior shoulder dislocation. Moreover, most of the primitive dislocation types belonged to the Neer two-part of GTF with shoulder dislocation type. A weak bone area may be present in the junction of the humeral head and shaft. Guo et al. [18] found that a positive relationship exists between the size of the greater tuberosity fragment and the occurrence of iatrogenic humeral neck fractures during the reduction of shoulder dislocation. In the case of elderly patients with primary shoulder dislocation, especially those together with GTF or occult anatomical neck fracture, the patients and their families should be informed of the potential risks in the course of reduction. Using a Kirschner wire in advance to reinforce the proximal humerus before reduction or ORIF directly is recommended [18].

For the choice of salvage operation for iatrogenic injuries, individualized treatment should be carried out depending on the patient’s age, fracture severity, and requirement of shoulder joint function [21, 22]. After the failure of manual reduction and the occurrence iatrogenic proximal humeral fracture, ORIF, even artificial shoulder arthroplasty and other rescue measures, should be prepared [23, 24]. In the present study, we all chose the rescue measures of ORIF and obtained clinical effects with different degrees of satisfaction. This result was attained partially because of the relatively young age of the patients, the complete mass fractures, the ideal reduction quality, and the reasonable functional rehabilitation after operation.

We also analyzed the relationship between the time from injury to revision surgery and the functional outcomes of ORIF. Unsurprisingly, those patients who had a treatment waiting time less than 8 h had better functional outcomes than those who waited more than 8 h. This significant difference demonstrates the importance of timely and effective ORIF for functional recovery. First, the blood supply of the anterior circumflex brachial artery branches have been destroyed after iatrogenic injury, and early operation to protect the posterior medial branch of the posterior circumflex brachial artery is the key to protecting the humeral head from ischemic necrosis. Second, the humerus head is in a non-physiological state for a long time, which increases the contact wear between the cartilage of the humeral head and the fracture section. Delayed ORIF likewise aggravated soft tissue edema and hemorrhage around the shoulder joint, followed by postoperative adhesions around the shoulder joint.

Our study is not without limitations. Specifically, the cases that include posterior dislocation are not involved. The reduction maneuver and number of reduction attempts are not described in the results, and the postoperative follow-up time is short, leading to certain limitations in postoperative functional results.

## Conclusion

The high-risk group of shoulder dislocation with GTF in senile females should be managed carefully. Effective reduction and internal fixation performed in a timely manner may help improve functional outcomes in the case of iatrogenic injury.

## Data Availability

The datasets used and/or analyzed during the current study are available from the corresponding author on reasonable request.
